# Freeze dehydration vs. supercooling of mesophyll cells: Impact of cell wall, cellular and tissue traits on the extent of water displacement

**DOI:** 10.1111/ppl.13793

**Published:** 2022-11-01

**Authors:** Matthias Stegner, Alexander Flörl, Jasmin Lindner, Sandra Plangger, Tanja Schaefernolte, Anna‐Lena Strasser, Viktoria Thoma, Janette Walde, Gilbert Neuner

**Affiliations:** ^1^ Department of Botany University of Innsbruck Innsbruck Austria; ^2^ Department of Statistics University of Innsbruck Innsbruck Austria

## Abstract

The extent of freeze dehydration of mesophyll cells in response to extracellular ice varies from supercooling to severe freezing cytorrhysis. The structural factors involved are poorly understood. In a comparison of mesophyll cells of 11 species, the factors “cell wall”, “cellular” and “tissue” traits were investigated. The extent of freeze dehydration was quantified as reduction in the sectional area during controlled freezing in the presence of ice. The cell wall thickness, cell size, cell area and the relative area of intercellular spaces were determined. The modulus of elasticity was determined by psychrometry. To grasp the relationships between factors and with freeze dehydration, we applied a principal component analysis. The first two components explain 84% of the variance in the dataset. The first principal component correlated negatively with the extent of freeze dehydration and relative area of intercellular spaces, and positively with the squared cell wall thickness to cell size ratio, elasticity and cell wall thickness. The cell size parameters determined the second principal component. Supercooling appeared preferable in cells with a high squared cell wall thickness to cell size ratio and a low relative area of intercellular spaces. Such factors are hypothesised to affect the magnitude of negative turgor pressure being built up below the turgor loss point. Negative turgor pressure slows dehydration by reducing the water potential gradient to the extracellular ice. With high levels of freeze dehydration, sufficient intercellular spaces for extracellular ice accommodation are needed. The low relative area of intercellular spaces increases cell‐to‐cell contact area and could support tissue stability.

## 1 INTRODUCTION

Freeze‐induced cellular changes and the associated functional processes are fundamental to understanding cryogenic injuries and frost survival of plants (Rajashekar & Burke, 1986). The mechanisms involved in freeze‐regulation of cells remain mostly unclear (Ishikawa et al., 2015; Ishikawa et al., 2018; Kishimoto et al., 2014). Knowledge of the biophysical mechanisms of how plant cells survive freezing (Wisniewski et al., 2014) is as important as the more extensively studied molecular mechanisms controlling changes to membrane lipids, sugars, osmolytes, proteins, genes and transcription factors (Knight & Knight, 2012; Pearce, 1999; Thomashow, 1999; Wisniewski et al., 2014). However, there is growing awareness of the important impact of cell wall structure and composition on plant freezing tolerance (Liu et al., 2022; Panter et al., 2019; Takahashi, Johnson, et al., 2021).

Ice‐tolerant plant cells survive exposure to a certain low freezing temperature, despite the formation of extracellular ice masses, while ice‐susceptible plant cells are killed upon ice formation in their tissues (Sakai & Larcher, 1987). Upon extracellular ice formation, ice‐tolerant cells either freeze‐dehydrate or resist the dehydrating forces and supercool. Mesophyll cells of ice‐susceptible species can accumulate extracellular ice that induces cell collapse (Ashworth & Pearce, 2002). Freeze dehydration is driven by a water potential gradient between the extracellular ice and the supercooled cell water, which is explained by the fact that the vapour pressure above water is higher than above ice. The displacement of cell water is strongly temperature‐dependent since the water potential gradient increases successively with decreasing freezing temperature (Beck et al., 1984; Hansen & Beck, 1988; Sakai & Larcher, 1987).

Freeze‐dehydration of cells results in cell shrinkage (Sakai & Larcher, 1987). The more moderate form has been termed freezing plasmolysis, that is, shrinkage of protoplast plus cell wall, and is fully reversible during thawing (Zhu & Beck, 1991). More severe freezing‐induced cell shrinkage due to freeze‐dehydration is called freezing cytorrhysis, in which cells partly collapse and the cell wall becomes deformed and wrinkled (Sakai & Larcher, 1987). When freeze‐dehydration follows the actual water potential gradient, ideal equilibrium freezing occurs (Olien, 1967). Less water movement towards the extracellular ice compared with what is expected from ideal equilibrium freezing results in a higher amount of symplastic water and consequently lesser ice masses; this is termed non‐ideal equilibrium freezing (Olien, 1967). In a study on *Citrus* leaves, cell wall rigidity was suggested to contribute to cold hardiness by resisting cell collapse and thus dehydration during extracellular freezing (Anderson et al., 1983). The positive effect of the plant cell wall on frost survival was experimentally proven by a study in which intact plant cells were compared with wall‐less isolated protoplast (Bartolo et al., 1987). Intact cells suffered from less frost injury than the respective wall‐less protoplast, showing that mechanical strain imposed by the cell wall during freeze–thaw stress is a major determinant of frost hardiness. Rigid cell walls allow a negative turgor pressure (Hansen & Beck, 1988; Rajashekar & Burke, 1986; Yang et al., 2017; Zhu et al., 1989; Zhu & Beck, 1991) that decreases the water potential gradient from liquid, supercooled cell water to extracellular ice and thus mitigates the extent of freeze dehydration (Rajashekar & Lafta, 1996). Cell wall rigidity has been shown to influence the extent of cell collapse (Pearce, 1988) and cell wall deformation (Fujikawa et al., 1999). Freezing cytorrhysis is a reversible process when the cell wall is elastic enough to allow contraction and expansion during freeze dehydration and rehydration upon thawing (Takahashi, Willick, et al., 2021).

For mesophyll cells, the extent of freeze‐dehydration can vary widely. Severe freeze‐dehydration has been demonstrated for *Sphagnum capillifolium* with a total approximated cell volume reduction of −81.8% at −5°C (Buchner & Neuner, 2010). In *Ranunculus glacialis* below −3°C, the cell area of mesophyll cells was reduced by about 50% (Stegner, Lackner, et al., 2020), and in mesophyll cells of *Hedera helix* at −8°C, <25% of cellular water remained unfrozen (Hansen & Beck, 1988). Ideal equilibrium freezing has been demonstrated for leaves (*Allium* bulb scales (Palta, Levitt, & Stadelmann, 1977), *Dendrosenecio keniodendron* (Beck et al., 1984), *Hedera helix* (Hansen & Beck, 1988)), crowns of cereals (Gusta et al., 1975) and red osier dogwood stem parenchyma (Harrison et al., 1978). However, freeze dehydration can be alleviated by rigid cell walls. Non‐ideal equilibrium was shown for leaves of *Citrus* (Anderson et al., 1983), *Hordeum vulgare* (Hansen & Beck, 1988) and *Pachysandra terminalis* (Zhu & Beck, 1991). This type of freezing increases at more severe frosts < −8°C and at high dehydration rates of 90% (Schulze et al., 2002), where the cell wall is pulled inwards and develops a tension instead of a pressure. During non‐ideal equilibrium freezing, higher amounts of water remain unfrozen in the protoplast (41–51% −10°C *Citrus* (Anderson et al., 1983), 30% −7°C *P. terminalis* (Zhu & Beck, 1991) than at ideal equilibrium freezing (15–25%). The most extreme form of non‐ideal equilibrium freezing is supercooling, in which little or no water is removed from the cells. Supercooling is reported for mesophyll cells of *Trachycarpus fortunei* (Larcher et al., 1991) and *Sasa senanensis* (Ishikawa et al., 2014). By a decreasing temperature, at some point supercooling can be no longer maintained, which results in intracellular freezing and cellular damage.

The identification of the main structural prerequisites of mesophyll cells and tissues that mitigate or promote freeze‐dehydration is still an open question: The extent of freeze‐dehydration is believed to be affected by factors like the cell wall rigidity, which is influenced by traits such as cell and tissue structure, intercellular spaces and cell size (Sakai & Larcher, 1987). Only few studies have examined the specific effect of cell wall rigidity on the extent of freeze‐dehydration (see previous paragraph). Most studies have directly linked cell wall properties to cold acclimation or increased cold hardiness without assessing the extent of freeze‐dehydration (Arias et al., 2015; Griffith et al., 1985; Griffith & Brown, 1982; Huner et al., 1981; Rajashekar & Lafta, 1996; Scholz et al., 2012; Stefanowska et al., 2002; Tanino et al., 2013; Weiser et al., 1990). The majority of studies focused on individual species; consequently, due to experiments conducted differently and the examination of different factors, the results are difficult to compare. Additionally, various methods have been used to quantify freeze‐dehydration: (1) by nuclear magnetic resonance and psychrometry, the percentage of unfrozen water in frozen tissues was measured (Anderson et al., 1983; Beck et al., 1984; Hansen & Beck, 1988; Palta, Levitt, Stadelmann, & Burke, 1977; Zhu & Beck, 1991). (2) Via differential thermal analysis, supercooling was detected (DTA, Larcher et al., 1991) and (3) by cryo‐microscopic methods, the change of cell volume during freeze dehydration has been quantified (Buchner & Neuner, 2010; Stegner, Lackner, et al., 2020).

The chemical components that increase the rigidity of cell walls in the mesophyll have hardly been addressed in view of freeze‐dehydration. Extensin was found to play an important structural role in the cell wall during cold acclimation of pea seedlings, perhaps by increasing rigidity of the cell wall and thereby increasing resistance to collapse caused by freeze‐induced dehydration (Weiser et al., 1990). In the supercooling species *T. fortunei*, silica deposition was thought to stiffen the cell walls (Larcher et al., 1991). In the mesophyll of *R. glacialis*, pectin‐rich cell walls with a relatively high esterification rate (55–70%) were discussed to aid the shifting of water to extracellular ice during cytorrhysis (Stegner, Lackner, et al., 2020). For supercooling *T. fortunei* leaves, small cells and narrow intercellular spaces, sclerenchyma richness and compartmentalization were hypothesised to be important (Larcher et al., 1991). Quite similar cytological and anatomical features are reported for the supercooling leaves of *S. senanensis* (Ishikawa et al., 2014). Extracellular ice accumulation in the mesophyll of *R. glacialis* was exclusively found in the spongy parenchyma, although palisade cells were freeze‐dehydrated to nearly a similar extent (Stegner, Lackner, et al., 2020). Strong freeze‐dehydration was observed for single cells and spongy tissues in *Pachysandra terminalis* (Zhu & Beck, 1991). These observations indicate specific tissue requirements for different degrees of freeze‐dehydration of mesophyll cells. A direct comparison of leaf types, which differ in their mesophyll cell and tissue architecture and their specific freeze‐dehydration response to extracellular ice, was not performed.

Our aim was to assess prominent structural factors influencing the extent of freeze dehydration in mesophyll cells of various plant species (evergreen vs. deciduous leaves, herbaceous vs. woody species) with a different tissue and leaf architecture. To the best of our knowledge, this is the first comprehensive study attempting to correlate cell wall, mesophyll cell and tissue characteristics with the extent of freeze‐dehydration at controlled freezing. We hypothesise that freeze‐dehydration of mesophyll cells is reduced for cells:

- with a high cell wall rigidity,
- with a high squared cell wall thickness to cell size ratio, a parameter that is indicative of a higher mechanical resistance and
- that are part of a tissue with a low intercellular space volume.

## 2 MATERIALS AND METHODS

### 2.1 Plant material

The freezing response of photosynthetically active mesophyll cells was studied in 11 higher plant species (Table 1). The species were chosen to include different morpho‐types of leaves, herbaceous and evergreen leaves, but also conifer needles. The maximum leaf freezing resistance of the studied species ranged between −3°C and −90°C (LT_50_). Only fully expanded leaves were collected randomly from three different locations: The Botanical Garden of University of Innsbruck (BG UIBK, 610 m a.s.l.), the Alpine Garden on Mt. Patscherkofel (AG UIBK, 1919 m a.s.l.) and on Mt. Kleiner Isidor (3185 m a.s.l.). *Solanum tuberosum* was cultivated in a field plot. *Citrus limon* leaves were collected from a potted tree that was integrated in a sand bed. *Galanthus nivalis*, *Leucojum vernum*, *Ranunculus glacialis*, *Vinca minor*, *Hedera helix*, *Picea abies*, *Pinus mugo* and P*inus cembra* were collected from natural growing sites. Potted individuals of *Trachycarpus fortunei* were cultivated in a greenhouse with temperatures between +10°C and +25°C. From species that show seasonally dependent frost hardening, the leaves were taken in the frost‐hardened state.

**TABLE 1:** The freezing response of mesophyll cells was studied in leaves of 11 higher plant species that differ in their leaf type, elevational range and the maximum freezing resistance

Species	Leaf type	Collection site	Coordinates	Maximum LT_50_ [°C]	Freezing behaviour
*Solanum tuberosum*	Herbaceous	BG UIBK field crop	47.26806 °N 11.38111 °E	−3^a^ ^,^ ^b^ ^,^ ^c^	Supercooling with intracellular freezing^a^
*Citrus limon*	Evergreen	BG UIBK greenhouse plant	47.26830 °N 11.38071 °E	−4^d^	Freeze dehydration non‐ideal equilibrium freezing^d^
*Galanthus nivalis*	Herbaceous	BG UIBK Cultivated and naturalised	47.26803 °N 11.37965 °E	−10^e^	Unknown
*Leucojum vernum*	Herbaceous	BG UIBK Cultivated and naturalised	47.26803 °N 11.37965 °E	−9^e^	Unknown
*Ranunculus glacialis*	Herbaceous	Mt. Kleiner Isidor natural growing site	46.97373 °N 11.10727 °E	−13^f^	Freeze dehydration^f^
*Vinca minor*	Evergreen	BG UIBK Cultivated and naturalised	47.26803 °N 11.37965 °E	−15^g^	Unknown
*Trachycarpus fortunei*	Evergreen	BG UIBK potted plant	47.26830 °N 11.38071 °E	−17^e^	Supercooling with intracellular freezing^h^
*Hedera helix*	Evergreen	BG UIBK Cultivated and naturalised	47.26803 °N 11.37965 °E	−25^g^	Freeze dehydration Ideal‐equilibrium freezing^i^
*Picea abies*	Evergreen needle	AG UIBK natural growing site	47.21089 °N 11.56055 °E	−50^j^	Unknown
*Pinus mugo*	Evergreen needle	AG UIBK natural growing site	47.21089 °N 11.56055 °E	−90^j^	Unknown
*Pinus cembra*	Evergreen needle	AG UIBK natural growing site	47.21089 °N 11.56055 °E	−90^j^	Unknown

*Note*: If something is already known about the species specific freezing behaviour, this is indicated under the point freezing behaviour.

^a^ Stegner et al. (2019).

^b^ Sakai and Larcher (1987).

^c^ Chen et al. (1976).

^d^ Anderson et al. (1983).

^e^ Unpublished Neuner Laboratory.

^f^ Stegner, Wagner, and Neuner (2020); Stegner, Lackner, et al. (2020).

^g^ Bauer et al. (1994).

^h^ Larcher et al. (1991).

^i^ Hansen and Beck (1988).

^j^ Sakai and Okada (1971).

The sampled leaves were detached and immediately stored in chilled thermal bags. The transportation time to the laboratory depended on the collection site but was not longer than 2 h, even for the nival site at 3185 m a.s.l. Maximum 4 days before the start of the experiments, the leaves were stored well‐watered in darkness at +4°C.

Mesophyll cells of *H. helix* (Hansen & Beck, 1988), *R. glacialis* (Stegner, Lackner, et al., 2020) and *G. nivalis* (Stegner, Wagner, & Neuner, 2020) perform freeze‐dehydration. Mesophyll cells of *C. limon* were reported to exhibit non‐ideal equilibrium freezing, although results were obtained at lethal freezing temperatures (Anderson et al., 1983). In a recent study, *S. tuberosum* cells could be shown to briefly supercool before they freeze intracellularly around −3°C (Stegner et al., 2019). Mesophyll cells of *T. fortunei* are known to persistently supercool (Larcher et al., 1991). For the other investigated species, the freezing response of their mesophyll cells was not known at the start of the study. The chosen species show structural differences in their mesophyll with respect to cell wall properties, cell and tissue types. The studied conifers (*Picea abies*, *Pinus cembra and Pinus mugo*) exhibit mesophyll cells of “arm palisade type” (Esau, 1977; Napp‐Zinn, 1966). Their cell walls have internal cell wall ridges projecting into the cell lumina.

### 2.2 Cell wall, mesophyll cell and tissue traits

#### 2.2.1 Anatomical parameters

Leaf anatomical and cell parameters were assessed by standard microscopical and stereological techniques on fresh leaf cross sections. Leaf cross sections were prepared from at least three mature leaves. The sampling of leaf pieces from the leaf blades used for sectioning was systematic and statistically random (Kubínová, 1993). A grid of dots (dot distance depended on the leaf size) was placed on the leaf blade. At each dot, rectangular leaf pieces were cut out with a scalpel. For conifer needles, needle pieces in different orientations were cut out at regular intervals. Since leaves are mostly not isotropic, the leaf pieces were cut out in three different angles to the main vein (0°, 45°, 90°). The leaf pieces were collected separately by angle in glass tubes filled with tap water. Sections (60–130 μm) were cut with a microtome (GSL1, Schenkung Dapples) from leaf pieces randomly picked. Analysis of cross sections was performed using a light microscope (BX50F, Olympus Corporation) coupled with a camera (DP25, Olympus Corporation). The software cell^D^ (Olympus Soft Imaging Solutions GmbH) was used to quantify the cellular and anatomical parameters. Per section, each parameter was measured 10 times, which in total yielded 30 measurements per parameter and cut angle. For simplification, we refer to the measured parameters as traits: The mesophyll cell wall thickness (*t*_CW_), cross‐sectional mesophyll cell area (*A*_*C*_), as well as width and height of mesophyll cells were averaged to cell size (*s*_*C*_) (Table 2). From *t*_CW_ and *s*_*C*_, a squared cell wall thickness to cell size ratio (*t*_CW_/*s*_*C*_)^2^ was calculated. The squared cell wall thickness to cell size ratio is similar to a factor called vessel implosion resistance, which was formerly used to describe the cavitation resistance of xylem vessels (Hacke et al., 2001; Jacobsen et al., 2005). The relative area of intercellular spaces (*R*_IC_) was calculated by dividing the intercellular area of leaf sections by the respective total leaf cross‐sectional area. Based on images taken from the sections, area fractions were measured with the software ImageJ 1.52a (Wayne Rasband, National Institutes of Health, Bethesda).

**TABLE 2:** List of parameters, anatomical features, and derived quantities, which are summarised to the term “traits”

Abbreviation	Trait	Unit
*A*_*C*_	Cell area	μm^2^
*s*_*C*_	Cell size = (mean width + mean height of mesophyll cells)/2	μm
*ε*	Modulus of elasticity	MPa
Ψ	Leaf water potential	MPa
*R*_❄red_	Extent of freeze dehydration − relative cell area reduction in the presence of ice	
*R*_IC_	Relative area of intercellular spaces	
RWC	Relative water content	
*t*_CW_	Mesophyll cell wall thickness	μm
(*t*_CW_/s_C_)^2^	Squared cell wall thickness to cell size ratio	
WSD	Water saturation deficit; that is, (1 − RWC)	

*Note*: Traits with their abbreviations and physical units are provided.

#### 2.2.2 Modulus of elasticity (ε)

“Pressure‐volume” curves provide information about plant‐water relations and particularly allow estimating ε. “Pressure‐volume” curves are obtained by recurrent measurements of leaf water potentials (Ψ) at full saturation and subsequent controlled dehydration. The leaf water potentials were measured with a PSYPRO Water Potential System (Wescor, Inc.) in C‐52‐SF Sample Chambers (Wescor, Inc.) with the PSYPRO Application Software (Wescor, Inc.). A two‐point calibration of the measurement chambers was realised with an osmolality standard (1000 mmol/kg NaCl; Opti‐Mole ELITechGroup) dropped onto filter paper discs. Prior to the leaf water potential measurements, the leaves were saturated by placing the petiole or, when still attached, the branches or stems for 12 h at +4°C in water wrapped up inside of a plastic bag. For leaf water potential measurements, leaf discs (Ø 7 mm) or leaf pieces (length 7 mm) were placed inside the C‐52‐SF sampler holders. To measure the saturation weight (SW), the mass of the samplers including the specimens was determined with a scale (Quintix65–1S, Sartorius Lab Instruments). Thereafter, the samplers including the specimen were sealed in the C‐52‐SF chambers. It took some time until water potential readouts were stable, as a vapour pressure equilibrium inside the samplers is required. Especially for conifer needles, it could last up to 24 h to reach equilibrium (Talbot et al., 1975). Only afterwards, the water potential measurements (Ψ) were reliably stable and could be taken. The PSYPRO Application Software allowed program‐repeated measurements and, consequently, to judge the species‐specific equilibration time. One by one, the specimen samplers were removed and immediately weighted to gain the fresh weight (FW) values. In case of short equilibrium times and unaffected viability, the specimens were inserted again into the C‐52‐SF chambers and the measurement procedure was repeated as long as the viability was not affected. This allowed monitoring the water potential relations throughout dehydration of the single specimens. This was not appropriate for species specimens, which needed a long time to equilibrate as the viability of cut samples can only be guaranteed for 2 days. In case of species with long equilibrium times, the water potential relations during dehydration were gained as follows: After determination of the SW, specimens were dehydrated at room temperature at moderate light conditions for unequal time periods until a desired state of dehydration was reached before they were sealed in the C‐52‐SF chambers and water potential was measured. After completion of the water potential measurements, the leaf samples were weighed again and dried in an oven (Heraeus T6060, Thermo Fisher Scientific Inc.) at 80°C for a minimum time period of 48 h. With the saturation weight (SW), the fresh weight (FW) and the dry weight (DW), the relative water content (RWC = (FW − DW)/(SW − DW)) and the water saturation deficit (WSD = 1 − RWC) were calculated. The described measurements were performed with viable leaves to measure the total water potential of the leaves ($\Psi_{\text{vital}}=\Psi_{\text{total}}$) and with freeze‐killed leaves to determine the osmotic water potential ($\Psi_{\text{non}-\text{vital}}=\Psi_{o}$), assuming a negligible matric potential (Tyree & Richter, 1981). For measurements on freeze‐killed leaves, right after determination of the saturation weight, the leaves were wrapped in Parafilm “M” (Pechiney Plastic Packaging) and aluminium foil, and dipped into liquid nitrogen for 30 s.

Pressure volume curves were obtained by plotting the negative inverse water potential (−1/Ψ) against the water saturation deficit (WSD). With the osmotic water potentials from killed leaves, a linear regression was calculated ($-1/\Psi_{\text{non}-\text{vital}}\sim \mathrm{WSD})$ to predict $\Psi_{o}\left(\mathrm{WSD}\right)$ over the whole WSD range. The predicted relationship of $\Psi_{o}\left(\mathrm{WSD}\right)$ was subtracted from the $\Psi_{\text{vital}}$ values to ascertain the turgor pressure ($\Psi_{P}$). To estimate the turgor pressure $\Psi_{P}\left(\mathrm{WSD}\right)$ as a function of the water saturation deficit, we used a modified exponential function (Schulte & Hinckley, 1985). The modulus of elasticity was obtained by derivation of the turgor pressure function ($\varepsilon \left(\mathrm{RWC}\right)=\frac{d\Psi_{P}}{d\mathrm{RWC}}\;\mathrm{RWC}$; Schulte & Hinckley, 1985). For the interspecific comparisons, we used the maximum elasticity at full turgor ($\varepsilon \left(\mathrm{RWC}=1\right)$). Note: A high elasticity value means high rigidity of the cells.

### 2.3 Quantification of the extent of freeze dehydration

The extent of freeze dehydration, that is, the relative cell area reduction in the presence of ice (*R*_❄red_), was quantified by cryo‐microscopic techniques at controlled cooling (Stegner, Lackner, et al., 2020). All the freezing experiments were conducted at precisely temperature‐controlled conditions. Therefore, a commercial freezer (GT series, Liebherr) was customised to be fully temperature controllable; for a detailed technical description, see Neuner et al. (2020). Air and leaf temperatures were recorded continuously with thermocouples (TT‐TI‐36 Omega Engineering Inc.). Freezing conditions were configured to be close to natural freezing conditions in the field; consequently, cooling and warming rates were limited to a maximum of 3 K h^−1^ (Arora, 2018; Neuner & Hacker, 2012).

For cryo‐microscopy, a light microscope (Leica DM1000, Leica Microsystems GmbH) was placed inside the cooling compartment of the temperature‐controlled freezer. The life image was captured by a camera (Leica EC4, Leica Microsystems GmbH) mounted on the microscope, which allowed monitoring of the leaf cross sections during controlled freezing. The software LAS EZ 3.0.0 (Leica Microsystems GmbH) was used for live view and image capture. An acrylic glass lid with integrated thermally insulated gloves on top of the freezing compartment enabled operating the microscope inside the freezer without affecting temperature. The leaf cross sections (60–130 μm) were placed in a water droplet under the coverslip on a microscope slide that was mounted at the cryo‐microscope stage. To prevent the sample from drying out and to trigger ice nucleation at a certain freezing temperature, a small bunch of moist cotton wool inoculated with ice nucleation active bacteria (*Pseudomonas syringae* van Hall 1902) was placed at the margin of the coverslip on the microscope slide. The bacteria initiated freezing between −2 and −3°C. From the cotton wool, a thin thread was elongated underneath the coverslip alongside the cross‐section. A thermocouple was installed next to the cotton wool to monitor sample temperature and to detect freezing. At regular intervals during the course of the freezing treatment, images were acquired for measurements of the cell area at the middle focal plane. For the respective samples, freezing temperatures were selected to not exceed the current state of freezing resistance; consequently, the experiments always happened at sub‐lethal temperatures. The cell area was determined by outlining cells with the freehand selection tool in ImageJ (Rasband, 1997‐2018). For each individual cell, the extent of freeze dehydration (*R*_❄red_) was calculated as a percentage of the cell cross‐sectional areas (*A*_*C*_) at +20°C and at the lowest sub‐lethal freezing temperature.

### 2.4 Data analysis

The correlations of the leaf traits (mesophyll cell wall thickness (*t*_CW_), mesophyll cell size (*s*_*C*_), cell area of mesophyll cells (*A*_*C*_), relative area of intercellular spaces (*R*_IC_), the modulus of elasticity (ε), extent of freeze dehydration (*R*_❄red_) and the freeze dehydration resistance factor (*t*_CW_/*s*_*C*_)^2^) together with the associations of the species were visualised by a principal component analysis (Table S1, PCA).

A multiple linear regression was computed to quantify and statistically test the association between *R*_❄red_ with two anatomical parameters (squared cell wall thickness to cell size ratio and relative area of intercellular spaces) and the functional parameter (modulus of elasticity):

(1) $$R{}_{❄\mathrm{red}}=\beta_{0}+\beta_{1}∙{\left(t_{\mathrm{CW}}/s_{C}\right)}^{2}+\beta_{2}∙\varepsilon +\beta_{3}∙R_{\mathrm{IC}}+u$$

where *β*_*i*_ (*i* = 0, … 3) are the regression coefficients and *u* is the remainder noise (Table S2).

Based on the assumption that (*t*_CW_/*s*_*C*_)^2^ relates to a vessel implosion resistance, which might negatively affect the extent of freeze dehydration, we included (*t*_CW_/*s*_*C*_)^2^ as a factor in our model. Furthermore, we hypothesise that *R*_IC_ affects *R*_❄red_ as intercellular spaces are a prerequisite for accommodating ice masses during freeze dehydration. As ε quantifies the rigidity of the plant tissue, we hypothesise that the extent of elasticity affects the extent of freeze dehydration. We did not include *s*_*C*_ and *t*_CW_ as traits as they are already included in the trait (*t*_CW_/*s*_*C*_)^2^. A_C_ was excluded due to multicollinearity considerations with *s*_*C*_. Multicollinearity was checked with the variance inflation factor (Belsley et al., 2005). Model assumptions were checked by residual diagnostics, a Shapiro–Wilk test for normality was used too. The dependent variable (*R*_❄red_) was continuous between 0 and 1, thus for a robustness check of the findings a beta‐regression was used (Table S3; (Cribari‐Neto & Zeileis, 2010)). The linear regression (Table S2) qualitatively provided the same results as the beta‐regression.^1^ Therefore, for the ease of interpretation, we discuss in the main text the findings of the linear regression. All the analysis were carried out in R (R Development Core Team, 2022). The significance level was set to 5%.

## 3 RESULTS

The mesophyll of the investigated species differed in cell wall, mesophyll cell and tissue traits (Table 3). The average cell wall thickness ranged from 0.6 μm (*L. vernum*/*V. minor*) to 2.0 μm in the needles of *P. abies*. The average cell size varied from 19 μm (*C. limon*) to 49 μm (*R. glacialis*). The mean cell area deviated greatly between the species from 374 μm^2^ (*T. fortunei*) to 2150 μm^2^ (*L. vernum*). The relative area of the intercellular spaces underlines differences in the mesophyll architecture. Intercellular spaces in the leaves of *S. tuberosum* and *T. fortunei* were below the resolution of the applied method. In contrast, *G. nivalis* leaves contain large air cavities that occupy 35 ± 5% in the sectional area. The modulus of elasticity ranged between 1.9 and 19.2 MPa; *Leucojum vernum* had the most elastic cells, whereas *T. fortunei* had the most rigid ones. Mesophyll cells of *C. limon*, *P. abies*, *P. cembra*, *P. mugo*, *S. tuberosum* and *T. fortunei* did not freeze‐dehydrate. The extent of freeze dehydration was about zero. In contrast, mesophyll cells of *G. nivalis*, *H. helix*, *L. vernum*, *R. glacialis* and *V. minor* showed freeze dehydration. Most extremely, mesophyll cell sectional area of *G. nivalis* was reduced by 75 ± 17%. Overall, six species were found to have supercooling mesophyll cells, including the most (*P. mugo*/*P. cembra*) and least (*S. tuberosum*) freezing resistant species. In the freeze‐dehydrating species group, *G. nivalis*, *L. vernum* and *R. glacialis* were present as herbaceous geophytes and *H. helix* and *V. minor* as perennial (woody) shrubs.

**TABLE 3:** Mean ± SD of cell wall thickness, cell size, cell area, the relative area of intercellular spaces and the extent of freeze dehydration obtained on mesophyll cells and the average modulus of elasticity of the 11 investigated species are provided

Species	Cell wall thickness (μm)	Cell size (μm)	Cell area (μm^2^)	Relative area of intercellular spaces (%)	Modulus of elasticity (MPa)	Extent of freeze dehydration (%)
*S. tuberosum*	0.9 ± 0.3	23 ± 8	1170 ± 353	0 ± 0	4.2	0 ± 0
*C. limon*	0.9 ± 0.3	19 ± 8	182 ± 57	4 ± 1	7.7	0 ± 0
*G. nivalis*	0.7 ± 0.3	29 ± 13	1180 ± 521	35 ± 5	3.0	75 ± 17
*L. vernum*	0.6 ± 0.2	41 ± 23	2150 ± 850	24 ± 6	1.9	66 ± 12
*R. glacialis*	1.0 ± 0.3	49 ± 19	2027 ± 574	13 ± 3	5.2	49 ± 14
*V. minor*	0.6 ± 0.1	22 ± 7	478 ± 129	6 ± 1	4.8	49 ± 12
*T. fortunei*	0.7 ± 0.1	22 ± 6	374 ± 92	0 ± 0	19.2	0 ± 6
*H. helix*	0.8 ± 0.2	27 ± 9	784 ± 224	7 ± 1	6.6	46 ± 38
*P. abies*	2.0 ± 0.5	48 ± 18	2045 ± 792	5 ± 6	13.6	1 ± 2
*P. mugo*	1.8 ± 0.4	43 ± 16	1681 ± 679	5 ± 5	15.5	0 ± 1
*P. cembra*	1.8 ± 0.5	41 ± 13	1642 ± 688	6 ± 6	6.7	0 ± 1

*Note*: *S. tuberosum* and *T. fortunei* have intercellulars spaces, but *R*_IC_ was <0.5%.

**FIGURE 1:**
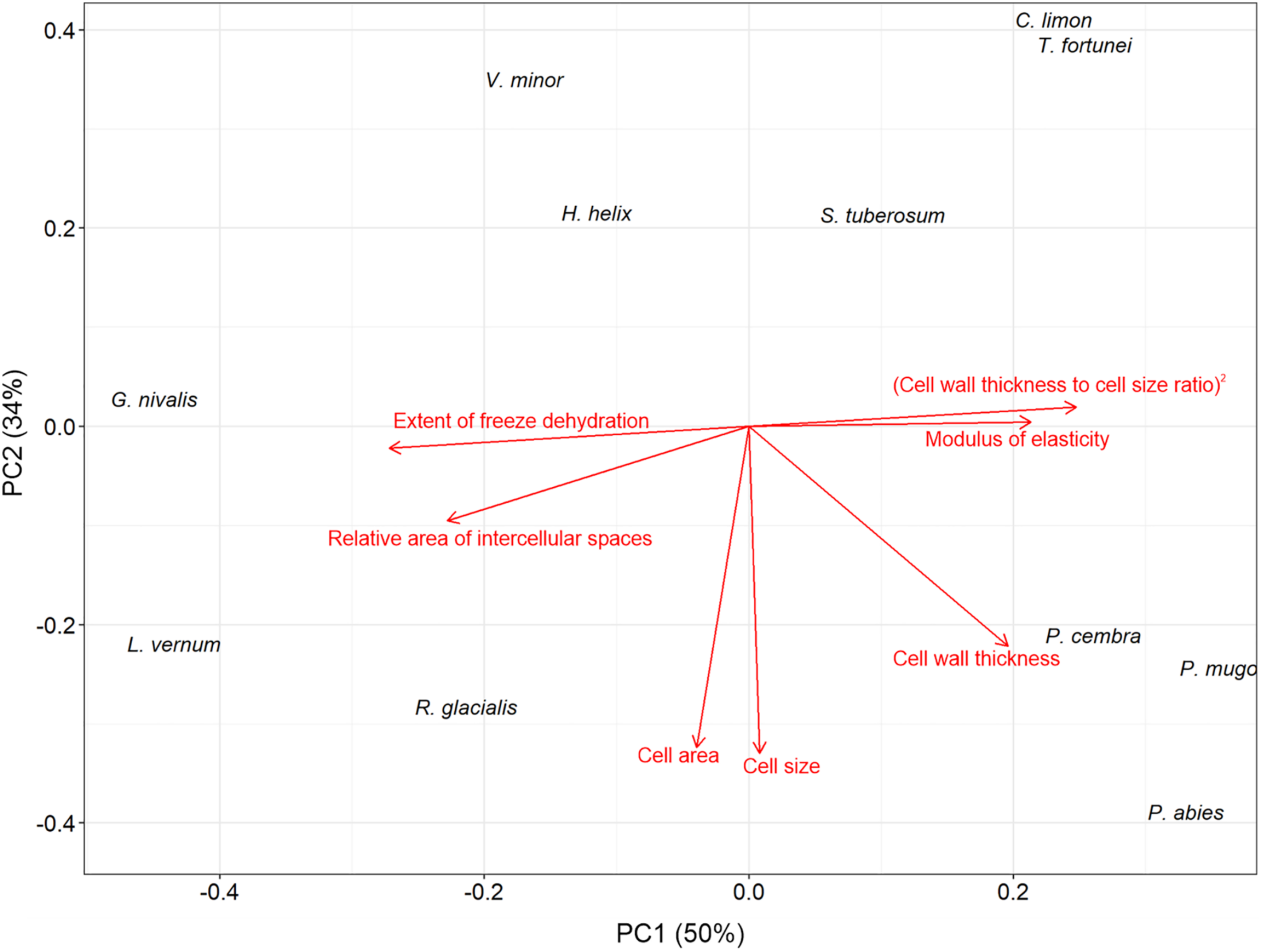
Principal component analysis with the seven leaf traits for 11 species. Loading plots for the first axis (explained variance 50%) and the second axis (explained variance 34%) are shown.

To describe similarities of the species with respect to the traits, the first two principal components explaining 84% of the total variance are used (Figure 1 and Table S1). The first component (PC1) correlated negatively with the traits “extent of freeze dehydration” and “relative area of intercellular spaces” but positively with the “squared cell wall thickness to cell size ratio”, the “modulus of elasticity” and the “cell wall thickness”. The second principal component (PC2) had a high negative correlation with the cell size and the cell area. Conifer needles were characterised by a high squared cell wall thickness to cell size ratio, rigid walls, did not freeze‐dehydrate and had a low relative area of intercellular spaces; interestingly, average cell size was high (>40 μm) (Figure 1). In contrast *G. nivalis*, *L. vernum* and *R. glacialis* freeze‐dehydrated, had a high relative area of intercellular spaces, but a low squared cell wall thickness to cell size ratio and elastic cell walls and were characterised by large cells. *Vinca minor* and *H. helix* had smaller cells, moderately freeze‐dehydrated and a relative area of intercellular spaces on average of 6% and 7%, respectively, moderately elastic cell walls and a low squared cell wall thickness to cell size ratio. *Citrus limon* and *T. fortunei* had small cells, a high squared cell wall thickness to cell size ratio, rigid cell walls and little intercellular spaces (<5%) and did not freeze‐dehydrate. *S. tuberosum* ranged somewhere between the group of *V. minor* and *H. helix*, and *C. limon* and *T. fortunei*.

The estimated linear regression to quantify the extent of freeze dehydration of the mesophyll cells with the traits explained 93% of the variance of *R*_❄red_ (*p* value <0.001, Table S3). The residual diagnostic supported the assumption of normality of the residuals (Figure S1; Shapiro–Wilk normality test, *p* value = 0.567), no multicollinearity was present (Table S4, largest variance inflation factor was smaller than 1.82), but the residuals were slightly heteroscedastic. Therefore, heteroscedastic robust standard errors were used (MacKinnon & White, 1985). The squared cell wall thickness to cell size ratio (*p* value = 0.004) and the relative area of intercellular spaces (*p* value = 0.044) significantly correlated with *R*_❄red_. The regression coefficient of the modulus of elasticity was statistically not significantly different from zero (*p* value = 0.336).

## 4 DISCUSSION

Modelling the extent of freeze‐dehydration yielded the squared cell wall thickness to cell size ratio and the relative area of intercellular spaces as significantly contributing traits (Table S3). The modulus of elasticity did not significantly correlate with the extent of freeze‐dehydration. However, this does not necessarily mean there is no effect as the sample size was quite small. Whether the modulus of elasticity correlates partially with the extent of freeze dehydration must remain the subject of future research. Furthermore, the fact that the modulus of elasticity integrates across all tissues of the leaf blade, and is not only a parameter of the mesophyll cells, might have alleviated its importance. Nevertheless, all these parameters importantly influence the extent of cellular turgor pressure, which is a major component of the total water potential of plant cells (Ψ_t_). Freeze‐dehydration of living plant cells is caused by a water potential gradient towards the extracellular ice masses. Below 0°C, the equilibrium pressure between ice and supercooled water decreases at a rate of −1.2 MPa K^−1^ (Beck et al., 1984). Consequently, keeping symplastic cellular water inside the cells in the presence of apoplastic ice masses must be considered as a big challenge.

### 4.1 Cell wall

Water can only be held inside cells if *Ψ*_*t*_ is sufficiently negative. Virtually two opportunities exist for the control of total cellular water potential (*Ψ*_*t*_), that is, osmotic (*Ψ*_*o*_) and turgor potential (*Ψ*_*p*_): $\left(-\right)\Psi_{t}=\left(-\right)\Psi_{o}+\left(\pm \right)\Psi_{p}$. During freeze‐dehydration, *Ψ*_*o*_ gets successively more negative as water content decreases. This is due to increasing solute concentration, which, however, would not suffice to compensate for the steep drop in water potential of ice with decreasing temperature as, for example, 1 mole of solutes causes the osmotic potential only to drop by −2.2 MPa (Körner, 2021). Short‐term adjustments of *Ψ*_*o*_ in higher plants do not exceed −1 MPa (Lösch, 2003) and, in response to seasonal cold acclimation, *Ψ*_*o*_ of leaves only dropped by −2.5 MPa at full saturation (Neuner et al., 1999). Our result obtained by pressure volume analysis indicates that even by a dehydration down to 50% WSD, *Ψ*_*o*_ does not drop by more than −0.8 to −1.6 MPa for freeze‐dehydrating cells. However, for supercooling mesophyll cells such as of *T. fortunei*, this could be −3.7 MPa (data not shown). Upon this, dehydration‐induced solute concentration and osmotic adjustments definitely mitigate freeze‐dehydration but cannot totally compensate for the applied water potential gradient at lower freezing temperatures. The second major determinant of *Ψ*_*t*_ is *Ψ*_*p*_. When a plant cell is saturated, *Ψ*_*p*_ is positive, but *Ψ*_*p*_ successively decreases with dehydration until the turgor‐loss‐point is reached (*Ψ*_*p*_ = 0). Below the turgor‐loss‐point, the rigidity of the cell wall importantly comes into play. Rigid cell walls can build up a significant negative cell wall tension that adds to *Ψ*_*o*_ and causes a more negative *Ψ*_*t*_ (Anderson et al., 1983; Zhu & Beck, 1991). Although repeatedly doubted (Rhizopoulou, 1997), experimental pieces of evidence for the existence of negative turgor pressure in leaf cells have been recently reviewed and the concept of negative turgor pressure in living cells seems generally accepted (Yang et al., 2017). Additionally, we have experimental data for the studied species that give evidence for a negative turgor pressure, particularly in tissues with rigid cell walls (Stegner and Neuner, unpublished). Our results suggest a decreased freeze‐dehydration of mesophyll cells that have a higher squared cell wall thickness to cell size ratio and less intercellular spaces. The squared cell wall thickness to cell size ratio indicates increased cell wall strength. The results found for mesophyll cells of conifer needles are in this line, the inwards oriented cell wall ridges of the arm palisade cells seem to be a structural component of the cell wall that allows building‐up further tension to increase negative *Ψ*_*p*_.

There is increasing awareness that cell wall structure and composition are important for plant freezing tolerance (Liu et al., 2022; Panter et al., 2019; Takahashi, Johnson, et al., 2021). The functional backgrounds of cell wall remodelling during cold acclimation are not really understood but include increased cell wall dry mass, increased soluble and insoluble galacturonic acid, increased pectin methylesterase activity and various changes in sugar composition (Liu et al., 2022). Specific roles and physical interactions of cellulose, xyloglucan and pectin that form the molecular basis of the complex mechanical behaviour of primary cell walls are reviewed by Cosgrove (2022). Cosgrove (2022) uncovers the dominant role of cellulose‐cellulose interactions in forming a strong yet extensible network and, strikingly, indentation stiffness of primary cell walls does not correspond to tensile stiffness, which appears significant in terms of freezing cytorrhysis. Of course, in the studied tissues, we are dealing with secondary cell walls that can contain even more stiffening chemical components such as minerals, lignin and suberin (Albersheim et al., 2010). For primary cell walls in particular, it has been hypothesised but not confirmed that endotransglucosylases, which contribute to xyloglucan integration, can promote wall loosening but also stiffening and are therefore now called wall‐remodelling enzymes (Cosgrove, 2022). Other potential wall‐stiffening effects have been suggested for pectin de‐esterification, but due to limited evidence for native cell walls, they appeared not a reliable indicator of cell wall mechanical behaviour (Cosgrove, 2022). Our results based on 11 species suggest that the cell wall traits responsible for negative tensions play an important role for freeze‐dehydration of cells, which has been also claimed earlier for single species (Anderson et al., 1983; Hansen & Beck, 1988; Rajashekar & Burke, 1986; Zhu et al., 1989; Zhu & Beck, 1991). Earlier, mainly cell wall porosity was discussed to be integral to intracellular freezing avoidance with the cell wall having ice barrier function (see Liu et al., 2022). However, an ice barrier function would only be relevant if intracellular freezing kills the cells and supercooling is the survival mechanism.

Nevertheless, without clear functional evidence, several observations found that cell wall rigidity, quantified by measurement of cell wall thickness or the modulus of elasticity, significantly increases during cold acclimation. Cell wall thickness (Griffith et al., 1985; Griffith & Brown, 1982; Huner et al., 1981; Stefanowska et al., 2002; Tanino et al., 2013; Weiser et al., 1990) and overall cell wall content (Panter et al., 2019) were found to increase during cold acclimation in cells of several different herbaceous species. After cold acclimation, the modulus of elasticity increased in leaves of broadleaf evergreen species (Rajashekar & Lafta, 1996), evergreen and deciduous cold desert shrub species (Arias et al., 2015; Scholz et al., 2012) but not in *Brassica napus* (Solecka et al., 2008). Also, ultrastructural differences were observed between the cell walls of different *Solanum* species with a frost‐resistant cultivar having a thicker cell wall than the ice‐susceptible one (Chen et al., 1976). For *Solanum sp*., intracellular freezing seems to be the frost‐killing event as mesophyll cells get frost damaged only during a second freezing exotherm (Stegner et al., 2019). *Arabidopsis* accessions that accumulated more cell wall material during cold acclimation were more freezing‐tolerant than those that accumulated less (Takahashi et al., 2019). All these studies showed that changes in the cell wall are relevant for increased freezing tolerance of mesophyll cells; our results offer a possible functional explanation.

### 4.2 Cell dimensions

The strongest parameter in PC1 was the squared cell wall thickness to cell size ratio, and in PC2 cell area and cell size. Strikingly, cell dimensions and cell wall attributes are hypothesised to be important feature for Ψ_p_: small cells were found to build up more negative pressure than large cells (Yang et al., 2017). Small cells and narrow intercellular spaces, sclerenchyma richness and compartmentalization were thought to be important for supercooling mesophyll cells of *Trachycarpus fortunei* (Larcher et al., 1991). Quite similar cytological and anatomical features are reported for leaves of other supercooling species such as other palms (Larcher & Winter, 1981), *Olea europea (*Larcher, 1970 *) Sasa senanensis* (Ishikawa et al., 2014), *Espeletia* (Goldstein et al., 1985; Larcher, 1975) and *Polylepis* (Rada et al., 1985). The structural requirements of supercooling are still hardly understood (Wisniewski et al., 2014). Our results obtained here indicate a prominent role of cell size and intercellular space volume. Recent results obtained for very frost‐hardy conifer needles suggest that they also might have supercooling mesophyll cells (Stegner and Neuner unpublished). Strikingly, conifer mesophyll cells are not small in size. We hypothesise that the cell wall architecture, with inwards oriented ridges (arm palisade type), compensates for the untypical large cell size otherwise not seen in “classical” supercooling tissues. This special cell wall structure must allow building‐up of strong negative tensions that alleviate freeze‐dehydration. Note that this may not be the case for all conifers. In a Cryo‐SEM study with *Pinus radiata*, mesophyll cells shrank during freezing, exhibiting freezing cytorrhysis (Roden et al., 2009). The species, however, is in contrast to the conifers studied here with a maximum hardiness of up to −90°C (USDA hardiness zone 1–4), only moderately frost‐hardy (−19°C USDA hardiness zone 8).

### 4.3 Intercellular space

In the applied model, the extent of freeze‐dehydration was also significantly affected by the relative area of intercellular spaces. Knowledge is scarce about tissue requirements for freezing cytorrhysis of cells, but two preconditions could be important: sufficient space for growing ice masses and additional tension built up by a high level of cell‐to‐cell contact. Sakai and Larcher (1987) report on persistently supercooling evergreen leaves, whose mesophyll cells do not show freezing cytorrhysis but keep the cell water supercooled. A common feature of these leaves was that they all had little, or hardly any, intercellular space. For *Pachysandra terminalis* leaves, strong dehydration of the tissue was typically seen for single cells and in intercellular rich spongy parenchyma (Zhu & Beck, 1991). For frozen wheat leaves, Pearce and Ashworth (1992) discuss that tissue structure (connections with adjacent cells), wall flexibility, and ice growth may all influence the shapes of the collapsing cells. The absence of intercellular spaces indicates that the middle lamella, which cements together the primary cell walls and is present in meristematic tissues, was not degraded during differentiation. Degradation of the middle lamella causes adjacent cells to separate, forming intercellular spaces. A tissue with few intercellular spaces may allow more relevant forces against cell collapse. The role of tissue compactness for freeze‐dehydration is not known but might be an important point when it comes to cell deformation.

Additionally, intercellular spaces are needed for the accommodation of extracellular ice when cells show a high extent of freeze dehydration. Generally, little is known about where in the extracellular space and how much ice forms in leaves during freezing nor how this is controlled. The intercellular spaces of the spongy tissue of the nival species *Ranunculus glacialis* were found to be completely filled with ice (Stegner, Lackner, et al., 2020). In other species, ice accumulation can occur in pre‐existing cavities, such as in leaves of *Galanthus nivalis* (Stegner, Wagner, & Neuner, 2020). Otherwise, it can also lead to reversible tissue displacements (Ball et al., 2004; Hacker & Neuner, 2007; Kaplenig et al., 2022; McCully et al., 2004; Schott et al., 2020) or, as in buds, even tissue disruptions (Kuprian et al., 2017). Nevertheless, water increases its volume during freezing. Whether the growing extracellular ice volume can develop a mechanical pressure on plant cells is not known.

The different extents of freeze‐dehydration observed for mesophyll cells during extracellular ice formation may allow the conclusion that there is a lack of a physiological reason for either. For both situations, physiological solutions have evolved. In any case, the herbaceous species studied are less frequently subjected to milder frosts than evergreens, and conifers still survive in sites with extreme and prolonged frosts and are among the most frost‐hardy species on earth. Whether the natural frost strain could be a driver for different extents of freeze‐dehydration observed for mesophyll cells is not known. When it comes to the ultimate limit of freezing temperatures, it long was thought that freeze‐dehydration is the better strategy as supercooling of mesophyll cells in leaves appeared limited down to about −21°C (Ishikawa et al., 2014). Very recent observations for conifer mesophyll cells may contradict this (Stegner et al., unpublished). Nevertheless, structural requirements for cells and tissues seem to exist when the extent of freeze‐dehydration of mesophyll cells is considered. These are namely intercellular space volume opposed to a strong connection of adjacent cells by an intact middle lamella and the squared cell wall thickness to cell size ratio. Consequently, the named cell and tissue characteristics and their interactions are all thought to influence negative turgor pressure and, by this, likely influence the shift of water and growth of extracellular ice during freezing.

## AUTHOR CONTRIBUTIONS

Gilbert Neuner designed the research. Matthias Stegner, Alexander Flörl, Jasmin Lindner, Sandra Plangger, Tanja Schaefernolte, Anna‐Lena Strasser and Viktoria Thoma performed the experiments and were responsible for data acquisition and analysis. Matthias Stegner combined the data and statistically analysed the dataset together with Janette Walde. Matthias Stegner and Gilbert Neuner wrote the draft of the manuscript, all authors carefully revised the manuscript.

## Supporting information


**TABLE S1**: Summary of the principal components analysis findings. With the first two principal components 84% of the total variance of the traits could be explained. Principal component 1 (PC1) was mainly positively correlated with the squared cell wall thickness to cell size ratio, the modulus of elasticity and the cell wall thickness, and negatively correlated with the extent of freeze dehydration and the relative area of intercellular spaces. PC2 was mainly determined by cell size and cell area.
**TABLE S2**: Linear regression analysis providing the associations of the squared cell wall thickness to cell size ratio (*t*_CW_/*s*_*C*_)^2^, the modulus of elasticity (ε) and the relative area of intercellular spaces (*R*_IC_) on the extent of freeze dehydration (*R*_❄red_). *Note*: *As residuals were slightly heteroscedastic robust standard errors were used.
**TABLE S3**: Beta‐regression analysis providing the associations of the squared cell wall thickness to cell size ratio (*t*_CW_/*s*_*C*_)^2^, the modulus of elasticity (ε) and the relative area of intercellular spaces (*R*_IC_) on the extent of freeze dehydration (*R*_❄red_). Note: A beta‐regression was used as robustness check because the dependent variable *R*_❄red_ was continuous between 0 and 1. Zero values were transformed accordingly by (*R*_❄red_ (*n* – 1) + 0.5/*n*), where n is the sample size.
**TABLE S4**: For multicollinearity diagnostics the variance inflation factors are provided. The relative area of intercellular spaces had the highest variance inflation factor followed by the squared cell wall thickness to cell size ratio and the modulus of elasticity but all of them were far below critical values known from the literature
**FIGURE S1**: Quantil‐quantil plot of the residuals for normality. The residuals of the linear model (Equation 1) are normally distributed according to residual diagnostics. Shapiro–Wilk test supported this conclusion. Note: Shapiro–Wilks test for normality supported the assumption of normally distributed residuals (*p* value = 0.567) but due to small sample size with small power for the alternative hypothesis.

## Data Availability

The data that support the findings of this study are available from the corresponding author upon request.

## ENDNOTE

^1^

The linear regression showed the same statistically significant traits and had similar high Pseudo‐Rsquares (=squared correlation between observed and estimated values) (cf. Tables S2 and  S3). The correlation between the estimates obtained by the linear regression and the ones by the beta‐regression was 0.99 and the mean absolute difference of both estimates only 0.04.
