# Supplementary material for: Freeze dehydration vs. supercooling of mesophyll cells: Impact of cell wall, cellular and tissue traits on the extent of water displacement
Source: Physiol Plant. 2022 Nov 1;174(6):e13793. doi: 10.1111/ppl.13793 (PMC9828361; doi:10.1111/ppl.13793)
Supplement: Supplementary file 1 — TABLE S1: Summary of the principal components analysis findings. With the first two principal components 84% of the total variance of the traits could be explained. Principal component 1 (PC1) was mainly positively correlated with the squared cell wall thickness to cell size ratio, the modulus of elasticity and the cell wall thickness, and negatively correlated with the extent of freeze dehydration and the relative area of intercellular spaces. PC2 was mainly determined by cell size and cell area. TABLE S2: Linear regression analysis providing the associations of the squared cell wall thickness to cell size ratio (t CW/s C )2, the modulus of elasticity (ε) and the relative area of intercellular spaces (R IC) on the extent of freeze dehydration (R ❄red). Note: *As residuals were slightly heteroscedastic robust standard errors were used. TABLE S3: Beta‐regression analysis providing the associations of the squared cell wall thickness to cell size ratio (t CW/s C )2, the modulus of elasticity (ε) and the relative area of intercellular spaces (R IC) on the extent of freeze dehydration (R ❄red). Note: A beta‐regression was used as robustness check because the dependent variable R ❄red was continuous between 0 and 1. Zero values were transformed accordingly by (R ❄red (n – 1) + 0.5/n), where n is the sample size. TABLE S4: For multicollinearity diagnostics the variance inflation factors are provided. The relative area of intercellular spaces had the highest variance inflation factor followed by the squared cell wall thickness to cell size ratio and the modulus of elasticity but all of them were far below critical values known from the literature FIGURE S1: Quantil‐quantil plot of the residuals for normality. The residuals of the linear model (Equation 1) are normally distributed according to residual diagnostics. Shapiro–Wilk test supported this conclusion. Note: Shapiro–Wilks test for normality supported the assumption of normally distributed residuals (p value = 0.567) b [file PPL-174-0-s001.docx]

Table S1: Summary of the principal components analysis findings. With the first two principal components 84% of the total variance of the traits could be explained. Principal component 1 (PC1) was mainly positively correlated with the squared cell wall thickness to cell size ratio, the modulus of elasticity and the cell wall thickness, and negatively correlated with the extent of freeze dehydration and the relative area of intercellular spaces. PC2 was mainly determined by cell size and cell area.

|  | Principal components | |
| --- | --- | --- |
| Traits | PC 1 | PC 2 |
| Cell wall thickness | 0.38 | −0.42 |
| Cell size | 0.02 | −0.63 |
| Cell area | −0.08 | −0.62 |
| Relative area of intercellular spaces | −0.44 | −0.18 |
| Modulus of elasticity | 0.41 | 0.01 |
| Extent of freeze dehydration | −0.52 | −0.04 |
| (Cell wall thickness to cell size ratio)^2^ | 0.47 | 0.04 |
|  |  |  |
| Standard deviation | 1.86 | 1.55 |
| Percentage of variance explained | 49.63 | 34.49 |
| Cumulative percentage of variance explained | 49.63 | 84.11 |

Table S2: Linear regression analysis providing the associations of the squared cell wall thickness to cell size ratio (t_CW_/s_C_)^2^, the modulus of elasticity (ε) and the relative area of intercellular spaces (R_IC_) on the extent of freeze dehydration (R󠇃_❄red_). Note: ^*^ As residuals were slightly heteroscedastic robust standard errors were used.

| Linear regression: R󠇃_❄red_ = β_0_ + β_1_∙(t_CW_/s_C_)^2^ + β_2_∙ε + β_3_∙R_IC_ + u | | | | | |
| --- | --- | --- | --- | --- | --- |
|  | Estimate | Std. Error | p-value | Robust^*^ Std. Error | Robust^*^ p-value |
| Intercept | 0.51 | 0.11 | 0.002 | 0.16 | 0.015 |
| (t_CW_/s_C_)^2^ | −244.31 | 55.33 | 0.003 | 58.92 | 0.004 |
| R_IC_ | 1.19 | 0.38 | 0.017 | 0.49 | 0.044 |
| ε | −0.0099 | 0.0064 | 0.180 | 0.0095 | 0.336 |
|  |  |  |  |  |  |
| Multiple R-squared | 0.93 | |  |  |  |
| Adjusted R-squared | 0.91 | |  |  |  |
| F-statistic | 33.25 on 3 and 7 DF | |  |  |  |
| p-value | < 0.001 | |  |  |  |

Table S3: Beta-regression analysis providing the associations of the squared cell wall thickness to cell size ratio (t_CW_/s_C_)^2^, the modulus of elasticity (ε) and the relative area of intercellular spaces (R_IC_) on the extent of freeze dehydration (R󠇃_❄red_). Note: A beta-regression was used as robustness check because the dependent variable R󠇃_❄red_ was continuous between 0 and 1. Zero values were transformed accordingly by (R󠇃_❄red_ּ (n-1)+0.5)/n, where n is the sample size.

| Beta-regression: R󠇃_❄red_ ~ B(µ, ɸ) with log(µ/(1− µ)) = α_0_ + α _1_∙(t_CW_/s_C_)^2^ + α _2_∙ε + α _3_∙R_IC_ | | | |
| --- | --- | --- | --- |
|  | Estimate | Std. Error | p-value |
| Intercept | 0.63 | 0.68 | 0.35 |
| (t_CW_/s_C_)^2^ | −1923.10 | 473.80 | < 0.001 |
| R_IC_ | 4.74 | 2.29 | 0.04 |
| ε | −0.06 | 0.06 | 0.27 |
| Log-likelihood | 18.87 on 5 DF | |  |
| Pseudo R-squared | 0.92 | |  |

Figure S1: Quantil-quantil plot of the residuals for normality. The residuals of the linear model (EQ 1) are normally distributed according to residual diagnostics. Shapiro-Wilk test supported this conclusion. Note: Shapiro-Wilks test for normality supported the assumption of normally distributed residuals (*p-value* = 0.567) but due to small sample size with small power for the alternative hypothesis.


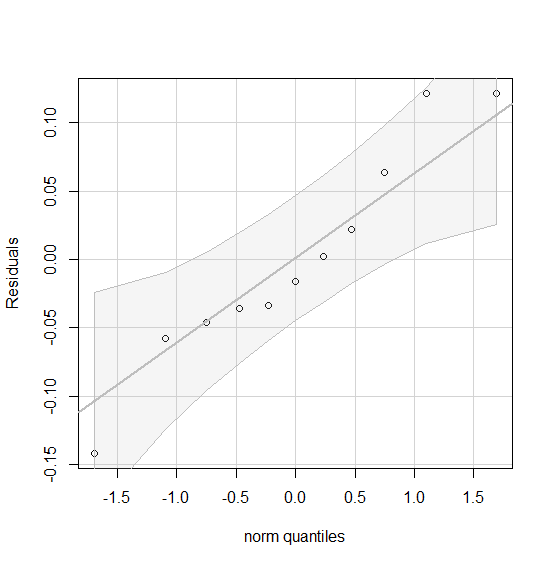


Table S4: For multicollinearity diagnostics the variance inflation factors are provided. The relative area of intercellular spaces had the highest variance inflation factor followed by the squared cell wall thickness to cell size ratio and the modulus of elasticity but all of them were far below critical values known from the literature.

| Trait | Variance inflation factor |
| --- | --- |
| R_IC_ | 1.82 |
| (t_CW_/s_C_)^2^ | 1.62 |
| ε | 1.53 |
